# Multi-Strain-Probiotic-Loaded Nanoparticles Reduced Colon Inflammation and Orchestrated the Expressions of Tight Junction, NLRP3 Inflammasome and Caspase-1 Genes in DSS-Induced Colitis Model

**DOI:** 10.3390/pharmaceutics14061183

**Published:** 2022-05-31

**Authors:** Abdullah Glil Alkushi, Sara T. Elazab, Ahmed Abdelfattah-Hassan, Hala Mahfouz, Gamal A. Salem, Nagwa I. Sheraiba, Eman A. A. Mohamed, Mai S. Attia, Eman S. El-Shetry, Ayman A. Saleh, Naser A. ElSawy, Doaa Ibrahim

**Affiliations:** 1 Department of Human Anatomy, Faculty of Medicine, Umm Al-Qura University, Al Abdeyah, Mecca 24382, Saudi Arabia; agkushi@uqu.edu.sa; 2 Department of Pharmacology, Faculty of Veterinary Medicine, Mansoura University, Mansoura 35516, Egypt; sarataha1@mans.edu.eg; 3 Department of Anatomy and Embryology, Faculty of Veterinary Medicine, Zagazig University, Zagazig 44511, Egypt; aabdelfattah@vet.zu.edu.eg; 4 Biomedical Sciences Program, University of Science and Technology, Zewail City of Science and Technology, October Gardens, 6th of October, Giza 12578, Egypt; 5 Department of Medical Biochemistry and Molecular Biology, Faculty of Medicine, Kafrelsheikh University, Kafrelsheikh 33516, Egypt; hala_ali2015@med.kfs.edu.eg; 6 Department of Pharmacology, Faculty of Veterinary Medicine, Zagazig University, Zagazig 44511, Egypt; gamal_vet_85@yahoo.com; 7 Department of Husbandry and Animal Wealth Development, Faculty of Veterinary Medicine, University of Sadat City, Sadat 32897, Egypt; nagwa.abosheraiba@vet.usc.edu.eg; 8 Department of Microbiology, Faculty of Veterinary Medicine, Zagazig University, Zagazig 44511, Egypt; emn.zewail@hotmail.com; 9 Zoology Department, Faculty of Science, Zagazig University, Zagazig 44511, Egypt; mai590259@gmail.com; 10 Department of Human Anatomy and Embryology, Faculty of Medicine, Zagazig University, Zagazig 44511, Egypt; emanelshetry@zu.edu.eg; 11 Department of Animal Wealth Development, Veterinary Genetics & Genetic Engineering, Faculty of Veterinary Medicine, Zagazig University, Zagazig 44519, Egypt; lateefsaleh@yahoo.com; 12 Department of Anatomy & Embryology, Faculty of Medicine, Zagazig University, Zagazig 44511, Egypt; naser_elsawy@ymail.com; 13 Department of Nutrition and Clinical Nutrition, Faculty of Veterinary Medicine, Zagazig University, Zagazig 44511, Egypt

**Keywords:** multi-strain probiotics, nanotherapy, colitis, tight junction, NLRP3 inflammasome, histopathological examination

## Abstract

Gut modulation by multi-strain probiotics (MSPs) is considered an effective strategy for treating inflammatory bowel disease (IBD). The combination of nanomaterial-based MSPs can improve their viability and resistance and can allow their targeted release in the gastrointestinal tract to be achieved. Thus, our aim is to investigate the prospective role of MSP integration into nanomaterials (MSPNPs) and the underlying molecular mechanisms supporting their application as an alternative therapy for IBD using a colitis rat model. To induce the colitis model, rats received 5% DSS, and the efficacy of disease progression after oral administration of MSPNPs was assessed by evaluating the severity of clinical signs, inflammatory response, expressions of tight-junction-related genes and NLRP3 inflammasome and caspase-1 genes, microbial composition and histopathological examination of colonic tissues. The oral administration of MSPNPs successfully alleviated the colonic damage induced by DSS as proved by the reduced severity of clinical signs and fecal calprotectin levels. Compared with the untreated DSS-induced control group, the high activities of colonic NO and MPO and serum CRP levels were prominently reduced in rats treated with MSPNPs. Of note, colonic inflammation in the group treated with MSPNPs was ameliorated by downstreaming NLRP3 inflammasome, caspase-1, *IL-18* and *IL-1β* expressions. After colitis onset, treatment with MSPNPs was more effective than that with free MSPs in restoring the expressions of tight-junction-related genes (upregulation of occludin, ZO-1, JAM, MUC and FABP-2) and beneficial gut microbiota. Interestingly, treatment with MSPNPs accelerated the healing of intestinal epithelium as detected in histopathological findings. In conclusion, the incorporation of MPSs into nanomaterials is recommended as a perspective strategy to overcome the challenges they face and augment their therapeutic role for treating of colitis.

## 1. Introduction

Ulcerative colitis (UC) and Crohn’s disease (CD) are two emerging sets of inflammatory bowel disease (IBD) and are mainly characterized by clinical symptoms such as loss of weight, abdominal pain, rectal bleeding and diarrhea [1]. The prevalence and incidence of IBD are growing in the world, with a higher rate among young adults, owing to modern lifestyle, hygiene, diet and industrialization [2,3]. Furthermore, patients that suffer from IBD for several years are at high risk of acquiring colitis-related cancer [4]. Genetic, immunological and environment factors are among the most causative agents of IBD. During the sequence of IBD, the damage of the intestinal epithelial barrier is considered to be the crucial event in the pathogenesis of IBD, which results in robust immune responses of the intestinal flora in a perspective of genetic predisposition [5]. In many inflammatory diseases, the over-activation of inflammasomes such as NLRP3 inflammasome is observed and results in the activation of caspase-1, which further induces inflammatory cytokine secretion, thereby controlling the inflammatory response [6]. A number of studies have indicated that NLRP3 inflammasome is closely associated with IBD remission [7,8]. Nutritional therapy has a promising role in maintaining IBD remission [9] by impacting intestinal inflammation via modifications of gut-microbiome antigen presentation and by boosting the mucosal immune system and epithelial barrier functions [10]. In this context, the oral administration of probiotics can exhibit significant potential as a natural alternative to unsuccessful long-term IBD treatment owing to their capacity to restore the homeostasis of gut microbiota, maintain gut barrier integrity, modify immune responses and give protection against invading pathogens, thus preventing chronic inflammation [11]. Multi-strain probiotics such as *lactobacillus*, *bacillus* and *bifidobacterium* or other mixtures are well-known beneficial probiotics that exert valuable effects through different mechanisms, including antibacterial compound production and the strengthening of gut epithelial barrier functions [12]. However, the effective functions of these probiotics’ combinations can be disrupted by adverse environmental and gastrointestinal conditions, thereby affecting their viability and colonization in the gastrointestinal tract. Thus, new approaches are necessary to maintain better cell stability and viability regardless of stomach acidity, avoiding their direct exposure to harmful environments. With the development of nanotherapy, searching for a nanocarrier system capable of delivering probiotics specifically and exclusively to the inflamed regions for a prolonged period of time is an unmet need. In this trend, the loading of probiotics into NPs can protect the active ingredients against degradation, improve their quality, bioavailability and safety and increase their stability [13]. Moreover, the mechanistic action of multi-strain probiotics on NLRP3 inflammasome activation is not yet understood. Thus, we prospect that using a combination of multi-strain probiotics double-coated with nanoparticles made from prebiotics could strengthen their long-lasting function in the remission and treatment of IBD. Particularly, if prebiotics such as chitosan and Na-alginate were developed into NPs, the resulting nanocomposites could have an additional valuable property such as the modulation of gut immune response or exert antimicrobial potential besides ensuring the arrival of probiotics to targeted sites and enhancing their circulating time inside the body. The purpose of this study is to synthesize a new innovative approach, for the first time, from multi-strain-probiotic-based NPs (MSPNPs) with better effect against IBD. Moreover, the curative and the precise molecular mechanisms of these MSPNPs against IBD via modulating the expressions of tight-junction-related genes and inflammatory cytokines, NLRP3 inflammasome and caspase-1 genes were investigated in the current study.

## 2. Materials and Methods

### 2.1. Preparation of Probiotic Microorganisms

Freeze-dried bacterial cells from *Lactobacillus acidophilus* (PTCC 4356) were rehydrated with 10 mL of MRS broth (de Man, Rogosa, Sharpe) (Oxoid, Thebarton, Australia) and incubated for at 37 °C for 24 h under aerobic conditions. For Bifidobacterium bifidum, cells were added to 18 mL of MRS broth, supplemented with 0.05% l-cysteine hydrochloride (Sigma, Thebarton, Australia) MMRS (Ventling and Mistry, 1993) to provide an anaerobic environment, at 37 °C for 48 h under anaerobic conditions using a Gas Pak system (Mitsubishi Gas Chemical Company Inc., Sydney, Australia) and then at 39 °C for 72 h under anaerobic conditions using the Gas Pak Plus system. For culturing Bacillus amyloliquefaciens CECT 5940, Luria Bertani (4 g/L sucrose, 5 g/L NaCl, 5 g/L tryptone and 10 g/L yeast, Oxoid Ltd., Basingstoke, UK) was used for 18 h at 32 °C. The cultures were transferred into MRS broth and incubated under the same conditions as before to obtain a cell density of about 1 × 10^10^ CFU/mL. The harvesting of cells was performed by centrifugation at 1500× *g* for 5 min at low temperature (4 °C) and the cell pellet was Bacterial strains comprising *Bifidobacterium bifidum*, *Lactobacillus acidophilus* and *Bacillus amyloliquefaciens* were supplied by Microbiology Lab., National Res. Centre, Dokki, Giza, Egypt.

### 2.2. Formulation and Characterization of Multi-Strain-Probiotic-Loaded Nanoparticles

**Figure 1:**
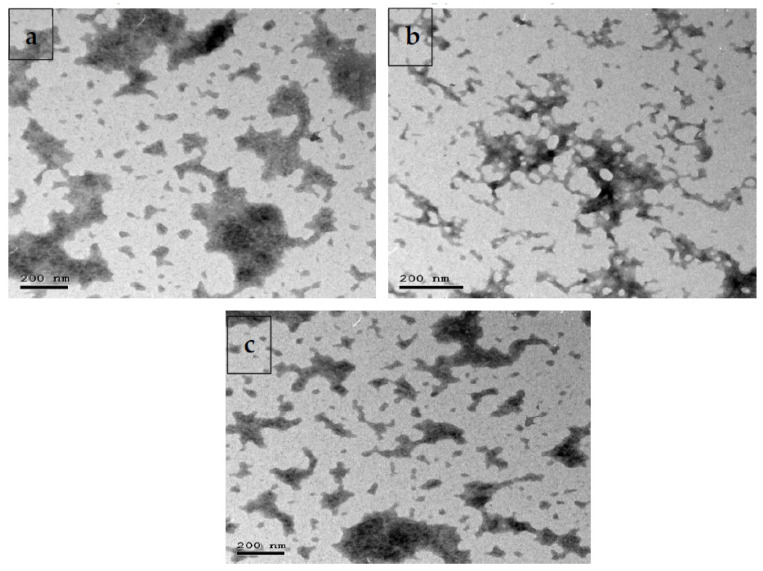
Transmission electron microscopy: nanoparticles loaded with *Lactobacillus acidophilus* (**a**) *Bacillus amyloliquefaciens* (**b**) and *Bifidobacterium bifidum* (**c**).

Probiotic bacteria including *Lactobacillus acidophilus*, *Bacillus amyloliquefaciens* and *Bifidobacterium bifidum* with chitosan aqueous solution (chitosan dissolved in 100 mL of distilled water and acidified with glacial acetic acid to attain 0.4% concentration (*w*/*v*); Sigma-Aldrich Saint Louis, MO, USA) were incorporated into 10 mL of 20 g/L sodium alginate (Sigma-Aldrich Saint Louis, MO, USA). After that, the prepared solution was filtered for eliminating insoluble material. The solution of sodium alginate was then added into the prepared chitosan solution and stirred to form beads. In the next step, these beads were strained from the chitosan solution and then rinsed with sterilized distilled water. Samples were dried under vacuum, with a freeze-drier (Freezedryer Lyobeta25). Dried cells were deposited in a dark place at 4 °C. The characterization of multi-strain-probiotic-loaded nanoparticles (MSP-NPs) was evaluated by transmission electron microscopy (TEM; Figure 1).

### 2.3. Survival of Assay of Probiotic-Cell-Loaded Nanoparticles in Bile Salt Solution and Incubation in Gastric and Intestinal Juice with and without Bile Salt

For testing the activity in bile salt: Freshly prepared MSPNPs (1 g) were added to a tube containing sterilized bile salt solution (10 mL; Oxoid, Thebarton, Australia) at pH 8.25, incubated for 2 h at 37 °C and then rinsed with 0.1% peptone solution. The viable cells of each bacterium were then counted on their selective media as described in Section 2.1.

For testing the activity in gastric and intestinal juice: As per the method described in [14], 10 mL of a suspension of activated sterile gastric juice (9 g/L of sodium chloride with 3.0 g/L of pepsin, pH adjusted to 2.0 with hydrochloric acid) and MSPNPs (1 × 10^10^ log cfu/g bead) was placed in a tube and incubated at 37 °C for intervals of 30, 60, 90 and 120 min. In the next step, free bacterial suspension was harvested and treated in sterile activated intestinal juice (9 mL; 0.835 g/L KCl, 6.5 g/L NaCl, 1.386 g/L NaHCO_3_ and 0.22 g/L CaCl_2_, pH 7.5 with or without 0.6% bile salt). In the next step, tubes were incubated for 150 min at 37 °C. After that, a 1 mL aliquot including dissolved free bacterial cells of each bacterium was taken from the activated intestinal juice and counted (CFU/mL) on tryptic soy broth for 24 h at 37 °C.

### 2.4. Experimental Animals and Colitis-Induced Model

All experimental animals were achieved in conformity with the guidelines and regulations authorized by the Institutional Animal Care and Use Committee (ZU-IACUC/f/90/2021), Faculty of Veterinary Medicine, Zagazig University. All animal protocols were carried out in agreement with the ARRIVE guidelines. Adult male Wistar rats (aged 7–8 weeks, 234 ± 17 g) were purchased from a laboratory animal house (Zagazig university). Prior to experiments, all rats were acclimated for two weeks before any experimental trial and kept under standard environmental conditions at a temperature of 23 ± 2 °C and humidity of 50 ± 3%; they received standard laboratory diet and water ad libitum. Dextran sodium sulfate (DSS) (5%, *w*/*v*) was used for the induction of the colitis model. According to the weight, experimental rats were randomly divided into four groups, i.e., a control group (non-colitic group; received standard diets and fresh drinking water and were orally gavaged for 7 days with PBS) and three DSS groups (colitic groups), in which rats received 5% DSS (*w*/*v*; molecular mass = M_w_ 7000–20,000; product No. 51227-100G; Sigma-Aldrich, Shanghai, China) dissolved in water ad libitum for 7 days, followed by oral gavage for another 14 days with either of the following: PBS (IBD control group); multi-strain probiotics (MSPs at a level of 1 × 10 ^10^ CFU/kg in 1 mL of PBS/rat/day); or MSPNPs (MSPNPs 1 × 10 ^10^ CFU/kg in 200 μL of PBS/rat/day). Over the experimental period, physical activity, clinical signs and body weight gain were observed daily. The severity of clinical signs was determined by assessing stool for the incidence of water or blood consistency. Colon and spleen were dissected for the evaluation of colon length and spleen weight. Gross rectal bleeding and stool consistency were estimated and scored according to standard procedures [15].

### 2.5. Sampling

At the end of the experimental trial, all experimental rats were anesthetized intravenously (30 mg/kg BW) with ketamine hydrochloride and then euthanized by cervical dislocation. Blood samples were collected for hematological profiling in heparinized tubes and for serum assortment (centrifuged for 10 min at 4 °C at 4000 rpm). Fecal samples were collected and used for the assessment of the fecal Calprotectin marker. Distal colon tissues (10 cm per portion) were collected, washed with PBS to eliminate any deposits and then divided into two sections. One sample was deposited quickly at −80 °C for further gene expression analyses. The other sample was fixed in 4% paraformaldehyde for eosin and hematoxylin (HE) staining.

### 2.6. Scoring by Disease Activity Index (DAI)

DAI scores are described in Table 1 and assessed from 0 (non-colitic and healthy) to 12 (severe colon inflammation) as previously stated by [16]. These scoring values were estimated for all animals, and the sum of three values was indicator of the DAI. For rectal bleeding evaluation, scoring varied from occult blood to gross bleeding. Moreover, diarrhea was proved by the manifestation of fecal mucus.

### 2.7. Measurement of Hematological and Serum Indices

Red blood cell (RBC) counts were established as specified by Brown (1976). Hemoglobin (Hb) concentration was quantified according to [17]. The serum concentrations of creatinine, urea alanine transaminase (ALT) and aspartate transaminase (AST) were calculated using standard kits (Sigma-Aldrich; MAK052, MAK055, MAK080 and MAK006, respectively). C-reactive protein (CRP) levels were evaluated according to the guidelines of a commercial kit (AG723-M; Sigma-Aldrich).

### 2.8. Measurement of Cytokines by Enzyme-Linked Immunosorbent Assay (ELISA)

For assessing the colon concentrations of IL-6, IL-10, TNF-α and *IFN-γ,* approximately 1 cm from colonic samples was cut into longitudinal segments and cleaned with PBS with penicillin/streptomycin. After that, serum-free RPMI 1640 medium with penicillin/streptomycin was utilized for the culturing of colon sections for 24 h. In the next step, cell-free supernatants were evaluated for cytokine secretion using Thermo Fisher cytokine ELISA kits (BMS625 BMS629, BMS622 and BMS621, respectively).

### 2.9. Measurement of Colonic Myeloperoxidase and Nitric Oxide

The neutrophil infiltration levels in colon tissues were evaluated by measuring Myeloperoxidase (MPO) activity as was identified by Krawisz, Sharon and Stenson [18]. Briefly, colonic samples were weighed and immediately homogenized in solution containing hexadecyl trimethyl ammonium bromide buffer; then, the prepared homogenates were centrifuged (25 min at 20,000 rpm), and the final supernatant (7 μL) was added to 200 μL of potassium phosphate buffer (pH 6.0) supplemented with O-dianisidine hydrochloride (0.167 mg/mL) and H_2_O_2_ (0.05 μL of 1%). Absorbance was determined at 450 nm. MPO results were quantified as units/gram (U/g) of tissue wet weight. Furthermore, colon homogenates were used for measuring total NO in the supernatant according to the methods described by Miranda, Espey and Wink [19]. Absorbance was calculated at 540 nm, and the results were expressed as nmol/g of colonic tissue.

### 2.10. Quantification of Fecal Calprotectin

The collected stool samples were mixed with extraction buffer and then homogenized for 25 min. After that, the homogenate was centrifuged for 20 min; then, the supernatant was frozen at −20 °C. Calprotectin was examined by enzyme-linked immunosorbent assay (ELISA; Thermo Fisher; MA5-12213) as described by Tøn, Brandsnes, Dale, Holtlund, Skuibina, Schjønsby and Johne [20].

### 2.11. Quantitative Real-Time PCR

Total RNA was extracted from colon samples using an RNeasy mini Kit (Qiagen, Valencia, CA, USA) as described in the manufacturer’s guidelines. The purity and quantity of extracted RNA were assessed using a NanoDrop™ ND-1000 Spectrophotometer (Thermo Fisher Scientific Inc., Waltham, MA, USA). cDNA was reverse-transcribed from the isolated total RNA mentioned above using a Maxima First-Strand cDNA Synthesis kit (Thermo Scientific, Waltham, MA, USA). The expressions of target mRNAs for genes encoding cytokines (interleukin (IL)-1β; tumor necrosis factor α (*TNFα*); *IL-6*, *IL-18* and *IL-10*; toll-like receptors (*TLR2* and *TLR9*); inflammasome-NLRP3; caspase-1) were quantified by real-time reverse-transcription polymerase chain reaction (qRT-PCR) using a Maxima SYBR green/ROX (6-carboxyl-X-rhodamine) qPCR Master Mix (Thermo Scientific). All PCR reactions were achieved in triplicate in a Stratagene MX3005P real-time PCR machine (Stratagene, La Jolla, CA, USA). The 2^–ΔΔCT^ method [21] was used for measuring relative gene expression levels, where β-actin was used as a control gene. The primer sequences targeting specific genes are shown in Table 2.

### 2.12. Quantitative DNA-Based Analysis of Abundance of Colon Bacterial Populations

Colonic contents were used for the extraction of total DNA using QIAamp Fast DNA Stool Mini (Qiagen, Hilden, Germany). The concentration and quality of previously extracted DNA were assessed using a Thermo Scientific NanoDrop 2000 spectrophotometer (Thermo Fisher Scientific Inc., Wilmington, DL, USA). Purified DNA samples were deposited at −80 °C for subsequent quantitative PCR evaluation. Real time PCR (RT-PCR) was finalized to calculate the targeted bacterial spp. abundance involving Bacillus, Bacteroides, Firmicutes, Enterobacteriaceae and Bifidobacterium, utilizing Stratagene MX3005P quantitative PCR. The targeted sequences of the primers of specific bacterial genes are described in Table 2. PCR amplification assessments were organized in triplicate, in 25 μL of reaction comprising 12.5 μL of SYBR Green PCR Master Mix (Qiagen, Hilden, Germany), 1 μL of each primer (10 mM), 9.5 μL of sterilized PCR-grade water and 2 μL of targeted genomic DNA. Standard curves were prepared with ten-fold serial dilutions of specific genomic DNA separated from pure bacterial cultures. In the next step, the standard calibration curves were created by plotting the threshold cycle (Ct) rates vs. the bacterial DNA copy records. The concentrations of bacteria in each DNA sample were counted by the given standard curves, in terms of the log_10_ CFU/g of the colonic contents. 

### 2.13. Histopathological Examination

Colon samples from the distal part were fixed in 10% neutral buffered formol saline for 24 h according to the previously published protocol [28,29]. After that, the tissue samples were washed and subsequently dehydrated in alcohol, embedded in paraffin (at 56 °C in hot air oven for 24 h) and hand-cut using a microtome (Leica). Then, cross sections of 5 μm were stained via the standard hematoxylin and eosin (HE) technique and evaluated under the microscopy [30]. The histological assessment of specimens was conducted to examine lesions such as epithelial damage, disrupted crypt architecture and infiltration of inflammatory cells [31,32].

### 2.14. Statistical Analysis

Statistical differences in the data were found using the one-way analysis of variance (ANOVA) in SPSS^®^ Statistics program version 22 (SPSS Inc., Chicago, IL, USA). The differences among groups were explored by Tukey’s post hoc tests. The normality of variance and homogeneity were evaluated by Shapiro–Wilk and Levene’s tests, respectively. All variables were conveyed as means ± standard error (SE). The minimal significant level was identified at *p*-values < 0.05. All graphs were made using GraphPad Prism software (Version 8, GraphPad Software Inc., San Diego, CA, USA).

## 3. Results

### 3.1. Survival of Free and Loaded Multi-Strain Probiotics in Activated Gastric and Intestinal Juice

The results marked that the reduction in the cell counts of free probiotic bacteria was more significant than that in nanoloaded probiotic bacteria. Of note, over 120 min, the initial counts of free Lactobacillus acidophilus, Bifidobacterium Bifidum and Bacillus Amyloliquefaciens were less stable and declined to 6.1 ± 0.22 × 10^5^, 5.1 ± 0.21 × 10^6^ and 6.1 ± 0.11 × 10^7^, respectively, when compared with the ones double-coated with nanoparticles, which were 5.8 ± 0.20 × 10^7^, 7.5 ± 0.29 × 10^7^ and 6.8 ± 0.22 × 10^8^ under the same stimulated gastrointestinal conditions (Table 3).

### 3.2. Effects of MSPNPs on Severity of Colon Injury

**Figure 2:**
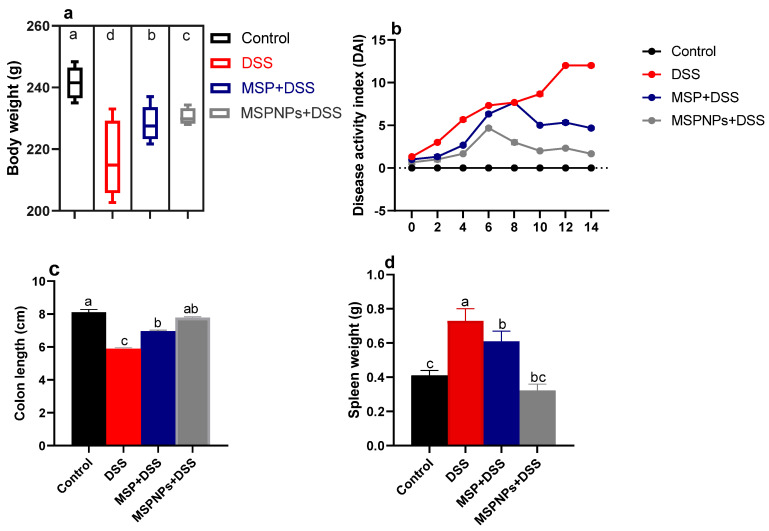
Impacts of orally administered free multi-strain probiotics (MSPs) or multi-strain-probiotic-loaded nanoparticles (MSPNPs) on progression of colitic signs. (**a**) Body weight gain. (**b**) Disease activity index score. (**c**) Colon length. (**d**) Spleen weight. Non-colitic group: Control (rats were orally gavaged with PBS). Colitic groups including: DSS (rats were orally gavaged with dextrane sodium sulphate), DSS + MSPs (rats were orally gavaged with DSS and multi-strain probiotics (MSPs) at the level of 1.0 × 10^10^ CFU/kg in 1 mL of PBS/rat/day for 14 days) and DSS + MSPNPs (rats were orally gavaged with DSS and multi-strain-probiotic-loaded nanoparticles (MSPNPs) at the level of 1.0 × 10^10^ CFU/kg in 1 mL of PBS/rat/day for 14 days); all groups were orally gavaged with 5% DSS. Values are expressed as mean ± SE, ^a,b,c,d^ Means of the bars with different letters were significantly different among groups (*p* < 0.05).

The effects of MSPNPs on ameliorating the severity of the clinical signs after the induction of colitis in rats are described in Figure 2. Notably, rats from DSS groups exhibited remarkable weight loss (up to 12%) compared with the 2.7% weight loss of the free-MSP-treated group (Figure 2a). Higher DAI scores were detected in the DSS-induced group due to an increased incidence of weight loss, diarrhea and rectal bleeding, while treatment with MSPNPs reversed the elevated DAI scores after the induction of colitis. Rats in the MSPNP-treated group showed the most significant (*p* < 0.05) colon length (8.1 ± 0.18 vs. DSS group of 5.88 ± 0.27 cm) (Figure 2b,c). Notable spleen enlargement was found in DSS colitic rats, while this enlargement was greatly reduced in the group treated with MSPNPs, unlike the non-colitic group (Figure 2d). All rats in the DSS group treated with MSPNPs were alive throughout the experimental period.

### 3.3. Hematological and Biochemical Estimation

The effects of MSPNPs on the hematological, and liver and kidney function tests are shown in Table 4. RBC counts were significantly (*p* < 0.05) lowered in both DSS-induced rats and DSS-induced rats treated with free MSPNPs. Meanwhile, colitic rats treated with MSPNPs showed no significant differences in RBC counts when compared with control non-colitic rats.

Moreover, the greatest reduction in Hb concentration (*p* < 0.05) was detected in the rats of the DSS group, while this reduction was reversed in the group treated with MSPNPs. The levels of AST and ALT in rats with DSS-induced colitis treated with MSPNPs were nearly similar to those in the control non-colitic group. Meanwhile, their levels were greatly increased in rats with DSS-induced colitis. Additionally, high levels of urea and creatinine after colitis induction were remarkably reduced in the DSS group of rats treated with MSPNPs. Markedly, the oral administration of MSPNPs after the induction of colitis maintained RBC counts and Hb concentrations to a large extent similar to those of the non-colitic group and recovered liver and kidney functions.

### 3.4. Assessment of Fecal Calprotectin Levels

**Figure 3:**
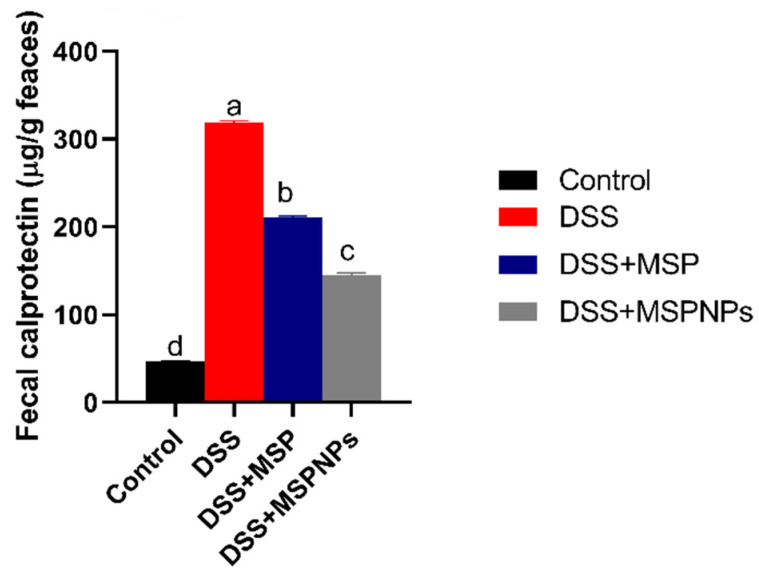
Effects of orally administered free multi-strain probiotics (MSPs) or multi-strain-probiotic-loaded nanoparticles (MSPNPs) on fecal calprotectin levels 14 days post DSS induction. Non-colitic group: Control (rats were orally gavaged with PBS). Colitic groups including: DSS (rats were orally gavaged with dextran sodium sulphate), DSS + MSPs (rats were orally gavaged with DSS and multi-strains probiotics (MSPs) at the level of 1.0 × 10^10^ CFU/kg in 1 mL of PBS/rat/day for 14 days), DSS + MSPNPs (rats were orally gavaged with DSS and multi-strain-probiotic-loaded nanoparticles (MSPNPs) at the level of 1.0 × 10^10^ CFU/kg in 1 mL of PBS/rat/day for 14 days); all groups were orally gavaged with 5% DSS. Values are expressed as mean ± SE, ^a,b,c,d^ Means of the bars with different letters were significantly different among groups (*p* < 0.05).

The levels of calprotectin in fecal samples after the induction of colitis are described in Figure 3. The fecal calprotectin levels in the colitic group were significantly (*p* < 0.05) elevated, while in the group treated with free MSPs, their levels were reduced. The prominent decline in fecal calprotectin levels was remarkably observed to be more pronounced in the MSPNP-treated group than in the free-MSP-treated group (*p* < 0.05) when compared with the colitic group.

### 3.5. Assessment of CRP Levels in Serum and of MPO and NO in Colonic Tissues

The levels of CRP were raised after DSS induction to 18.39 and only to 10.36 and 4.33 U/g in the groups that received free MSPs and MSPNPs, respectively, when compared with the non-colitic group. The highest activities (*p* < 0.05) of MPO in colon tissues were observed in colitic groups (14.36 U/g), while this elevated level was markedly reduced in the group treated with MSPNPs (Table 4). Remarkably, DSS induction elevated the levels of NO, in contrast to the treatment with MSPNPs, which greatly reduced (*p* < 0.05) its levels.

### 3.6. Quantification of Inflammatory Mediators in Colon by ELISA and Real-Time PCR

**Figure 4:**
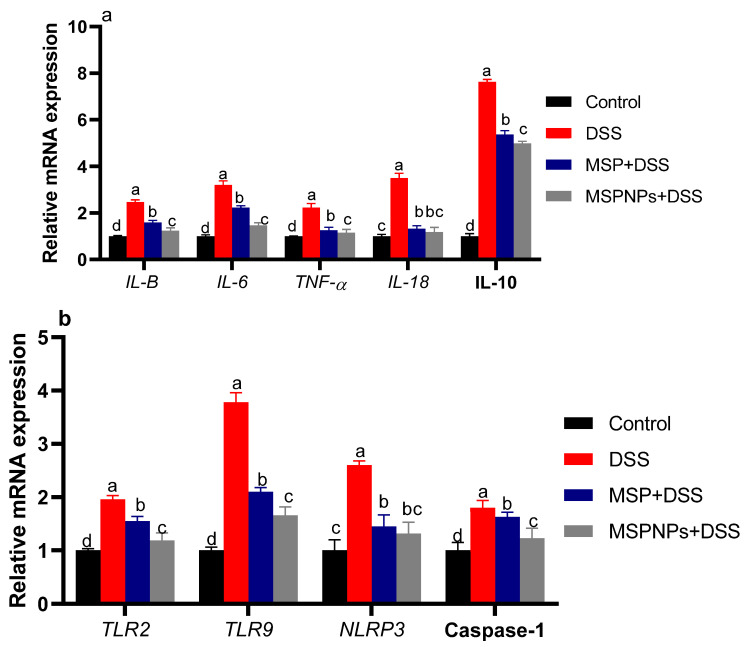
Effects of orally administered free multi-strain probiotics (MSPs) or multi-strain-probiotic-loaded nanoparticles (MSPNPs) on: (**a**) mRNA expressions of cytokine-related genes, Interleukin (IL)-1β, IL-6, IL-18, IL-10 and tumor necrosis factor α (TNFα); (**b**) expressions of toll-like receptors (TLR2 and TLR9) pyrin domain-containing protein 3 (NLRP3) inflammasome and caspase-1 at 14 days post DSS induction. Non-colitic group: Control (rats were orally gavaged with PBS). Colitic groups including: DSS (rats were orally gavaged with dextran sodium sulphate), DSS + MSPs (rats were orally gavaged with DSS and multi-strains probiotics (MSPs) at the level of 1.0 × 10^10^ CFU/kg in 1 mL of PBS/rat/day for 14 days), DSS + MSPNPs (rats were orally gavaged with DSS and multi-strain-probiotic-loaded nanoparticles (MSPNPs) at the level of 1.0 × 10^10^ CFU/kg in 1 mL of PBS/rat/day for 14 days); all groups were orally gavaged with 5% DSS. Values are expressed as mean ± SE. ^a,b,c,d^ Means of the bars with different letters were significantly different among groups (*p* < 0.05).

The inflammatory response in the colon was assessed by measuring the levels of IL-6, TNF-α, IFN-γ and IL-10 cytokines using ELISA kits, as shown in Table 4. The highest inflammatory response (*p* < 0.05) was perceived in the colitic non-treated group, as proved by the remarkable increase in pro-inflammatory cytokines TNF-α, IFN-γ and IL-6 and anti-inflammatory IL-10 levels. Meanwhile, the levels of these inflammatory cytokines were lowered in the group that received MSPNPs, followed by the group that received free MSPs. Additionally, the inflammatory response in the colon was further confirmed by analyzing the expression levels of pro-inflammatory-cytokine-related genes and anti-inflammatory IL-10 genes in colon tissues (Figure 4). It was remarkable that rats that suffered from DSS-induced colitis had the highest expression levels of pro-inflammatory cytokines IL-6, IL-1β and TNF-α and anti-inflammatory IL-10 levels. On the other hand, MSPNPs markedly reduced cytokine levels, which indicates their role in the recovery from induced inflammation. The suppression of colitis by MSPNPs was further investigated by assessing the expression levels of NLRP3 inflammasome and caspase-1 (Figure 4). Interestingly, the upregulated levels of NLRP3 inflammasome and caspase-1 noticed in the colonic mucosa of colitic rats (increased by 1.45 and 1.63, respectively) were more prominently expressed at low levels, especially in groups treated with MSPNPs.

Moreover, a significant upregulation of TLR2 and TLR9 was observed in the group with active colitis. Of note, their excessive expression levels were significantly reduced in the group treated with MSPNPs more than in the group treated with free MSPs after colitis induction.

### 3.7. Tight Junction Protein Expression in Colitic Rats

**Figure 5:**
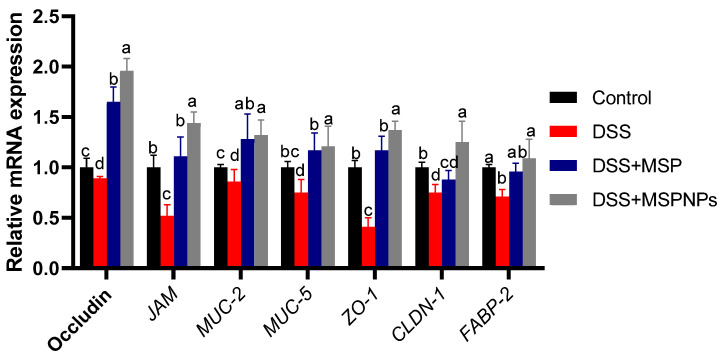
Effects of orally administered free multi-strain probiotics (MSPs) or multi-strain-probiotic-loaded nanoparticles (MSPNPs) on mRNA expressions of tight-junction-related genes (occludin, junction adhesion molecule (JAM), mucin-2 (MUC-2), mucin-5 (MUC-5), zonula occludens (ZO-1), claudins (CLDN-1) and fatty acid binding protein (FABP-2)) 14 days post DSS induction. Non-colitic group: Control (rats were orally gavaged with PBS). Colitic groups including: DSS (rats were orally gavaged with dextran sodium sulphate), DSS + MSPs (rats were orally gavaged with DSS and multi-strains probiotics (MSPs) at the level of 1.0 × 10^10^ CFU/kg in 1 mL of PBS/rat/day for 14 days), DSS + MSPNPs (rats were orally gavaged with DSS and multi-strain-probiotic-loaded nanoparticles (MSPNPs) at the level of 1.0 × 10^10^ CFU/kg in 1 mL of PBS/rat/day for 14 days); all groups were orally gavaged with 5% DSS. Values are expressed as mean ± SE. ^a,b,c,d^ Means of the bars with different letters were significantly different among groups (*p* < 0.05).

As shown in Figure 5, following DSS exposure, the expressions of tight-junction-related genes were markedly reduced in the DSS-induced group when compared with the control non-colitic group. Meanwhile, the expressions of occludin, JAM, ZO-1 and CLDN-1 reached their peak in the colitic group that received MSPNPs (increased 1.96-, 1.44-, 1.37- and 1.25-fold vs. control non-colitic group). The high expressions of MUC-2 and MUC-5 were more prominent in the group that received MSPNPs followed by the group that received free MSPs when compared with the control non-colitic group (increased 1.32-, 1.37-, 1.28- and 1.17-fold, respectively). On the other hand, the expression of FABP-2 in groups treated with either free MSPs or MSPNPs exhibited no statistical differences when compared with the control non-colitic group.

### 3.8. Analysis of Microbial Populations in Colon Contents

**Figure 6:**
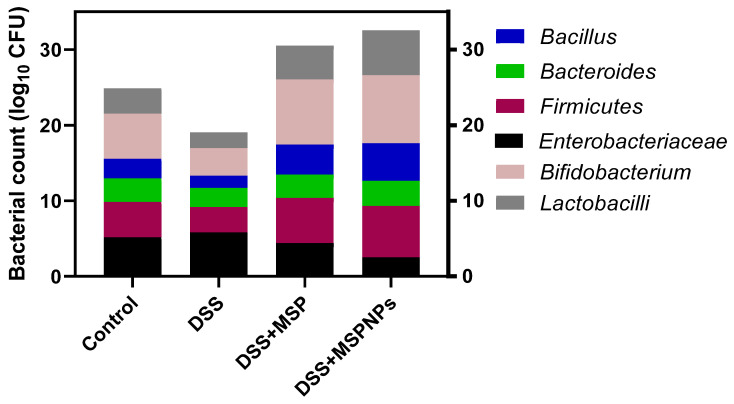
Effects of orally administered multi-strain probiotics (MSPs) or multi-strain-probiotic-loaded nanoparticles (MSPNPs) on abundance of *Bacillus*, *Bacteroides*, *Firmicutes* and *Enterobacteriaceae Bifidobacterium* populations and *lactobacilli* (log_10_ CFU), 7 days post DSS induction. Non-colitic group: Control (rats were orally gavaged with PBS). Colitic groups including: DSS (rats were orally gavaged with dextran sodium sulphate), DSS + MSPs (rats were orally gavaged with DSS and multi-strains probiotics (MSPs at the level of 1.0 × 10^10^ CFU/kg in 1 mL of PBS/rat/day for 14 days), DSS + MSPNPs (rats were orally gavaged with DSS and multi-strain-probiotic-loaded nanoparticles (MSPNPs) at the level of 1.0 × 10^10^ CFU/kg in 1 mL of PBS/rat/day for 14 days); all groups were orally gavaged with 5% DSS.

The efficacy of orally administered MSPs or MSPNPs on the counts of bacterial populations in the colon are displayed in Figure 6. Notably, the colitic groups that received MSPs or MSPNPs showed unique alterations in the microbial population when compared with other groups (control or DSS groups). The abundance of beneficial bacterial populations was considerably increased; on the contrary, the abundance counts of the pathogenic ones were significantly reduced. Remarkably, compared with the DSS group, the colitic group that received MSPNPs showed the highest Firmicutes abundance. Furthermore, the counts of beneficial bacterial populations (Bacillus, Lactobacillus and Bifidobacterium) were assessed in the colitic groups that received MSPNPs (4.96, 5.97 and 8.97 CFU/g) and free MSPs (3.97, 4.48 and 8.60 CFU/g) and compared with the DSS-induced group (2.59, 3.37 and 3.67, respectively). Moreover, the population of Enterobacteriaceae was more prominently reduced in the colitic group that received MSPNPs.

### 3.9. Histological Changes in the Colon

**Figure 7:**
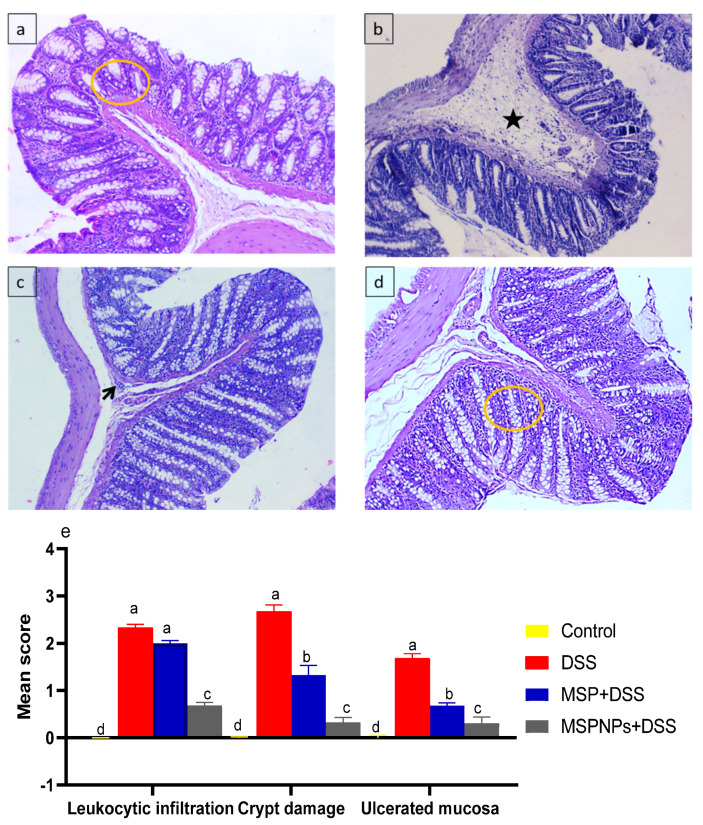
Histological changes following oral administration of multi-strain probiotics (MSPs) or multi-strain-probiotic-loaded nanoparticles (MSPNPs) in colitic rats. Non-colitic group: Control (rats were orally gavaged with PBS) (**a**) Colitic groups including: DSS (rats were orally gavaged with dextran sodium sulphate) (**b**) DSS + MSPs (rats were orally gavaged with DSS and multi-strains probiotics (MSPs) at the level of 1.0 × 10^10^ CFU/kg in 1 mL of PBS/rat/day for 14 days) (**c**) DSS + MSPNPs (rats were orally gavaged with DSS and multi-strain-probiotic-loaded nanoparticles (MSPNPs) at the level of 1.0 × 10^10^ CFU/kg in 1 mL of PBS/rat/day for 14 days) (**d**) all groups were orally gavaged with 5% DSS. Yellow circle: normal colon crypt structure. Arrowhead: ulcerated mucosa with abundant leukocytic infiltrations within lamina propria. Star: presence of serofibrinous exudate within submucosa. Scoring criteria (**e**) 0 = no; 1 = mild; 2 = moderate; and 3 = severe lesion. Scoring was performed blindly by an independent pathologist. ^a,b,c,d^ Means of the bars with different letters were significantly different among groups (*p* < 0.05).

The histological findings of the colon samples of healthy rats (non-colitic, control group) showed no damage to the crypt architecture and normal columnar epithelium of the mucosa with numerous goblet cells, lamina propria, submucosa and muscular layer with no sign of leukocytic infiltration (Figure 7a). In the DSS-induced colitic group, we found an ulcerated mucosa with widespread leukocytic infiltrations (inflammatory-cell infiltration) within the lamina propria (arrowhead) beside the presence of serofibrinous exudate within the submucosa (Figure 7b). In contrast, in the group treated with MSPs, the severity of the lesion was much reduced, as they showed an apparently normal mucosa, and impacted submucosal blood vessels with round cells were detected (Figure 7c). Moreover, the group treated with MSPNPs showed the best amelioration among all colitic groups, as the preserved cytoarchitectures of mucosa, submucosa, muscularis and serosa were observed (Figure 7d).

## 4. Discussion

Dietary modulations focused on the gut microenvironment are considered as novel therapies to cure functional bowel disorders that mostly aim at modifying the gut microbiota [32,33]. The administration of multi-strain probiotics such as *Lactobacillus*, *bacillus* and *Bifidobacterium* has been proved to have beneficial impacts on mouse models suffering from colitis [34]. New insights are directed toward the incorporation of probiotics strains into nanoparticles in order to increase their survival and viability in the gastro-intestinal environment. Prospective applications of new formulations of prebiotic nanoparticles with probiotics strains have not only increased their antimicrobial activity but also protected them from gastrointestinal conditions [35]. Recently, Alkushi [28] described that the application of *bacillus amyloliquefaciensin* in nanoform could attenuate the severity of DSS-induced colitis. However, the administration of multi-strain probiotics incorporated into nanoparticles for curing IBD have not yet been investigated. In the present study, the results indicate, for the first time, that MSPNPs exerted protective effects in experimental rat models with DSS-induced colitis. Herein, it was notable that DSS-induced colitic rats displayed higher DAI scores as marked by the decrease in body weight that resulted from loss of appetite, nutrient malabsorption and diarrhea accompanied by colorectal bleeding. Additionally, a decrease in colon length with marked enlargement of the spleen was detected in the colitic non-treated group. Likewise, DSS-induced mice showed, on day 5, prominent weight loss (around 5–10% reduction) and changed stool consistency as consequences of bloody diarrhea [36]. In contrast, the oral administration of MSPNPs improved the general conditions of DSS-treated rats more than free MPSs, as remarked by the attenuation of body weight loss and enlargement of spleen weight, as well as an increase in colon length. In accordance, mice fed microencapsulated *Ligilactobacillus salivarius* displayed greater colon length unlike mice with DSS-induced colitis [37].

MPO activity is a good indicator of leucocyte infiltration and a sensitive marker for inflammatory reactions in various tissues [38]. In the current study, the influx of inflammatory cells into the injured colon was prominent in the colitic group and was accompanied by increased colonic MPO activity, which is in accordance with Ali et al.’s results [39]. Moreover, the severity of colitis is associated with higher levels of serum CRP, which is a biomarker of colon inflammation [40]. Interestingly, the damage associated with colitis resulted in a significant elevation of serum levels of CRP and MPO activity in the colon, while these elevated levels were notably reduced in those rats that received MSPNP treatment, which exerted their protective role against colon damage. In accordance, after treatment with probiotic mixtures comprising *Lactobacillus delbruekii* and *Lactobacillus fermentum,* a decreased colonic activity of MPO was observed [41]. Additionally, remarkable oxidative stress was noticed after the induction of colitis, as reflected by higher NO levels in colon tissues. Meanwhile, the protective effects of MSPNPs against oxidative stress were marked by lowered NO levels in colonic tissues. This can be attributed to the potential antioxidant mechanisms of probiotics, including the production of antioxidant metabolites and decrease in enzyme activities that mediate the production of ROS [42]. All these explanations could strengthen the role of MSPNPs in lessening the risk of colon inflammation, in agreement with Celiberto et al. [43]. Fecal calprotectin (FC) is a complex cytosolic protein, commonly found in neutrophils, macrophages and epithelial cells, including intestinal epithelial cells [44]. Besides, it exerts an antimicrobial impact by binding to calcium and is considered as evident throughout cell activation or death [45]. The levels of FC are significantly elevated in numerous inflammatory processes, and it is considered as a diagnostic biomarker of disease severity, especially in IBD [46]. FC is usually supposed to be a pro-inflammatory protein that plays the role of alarm in inflammatory sites through the activation of the receptor for advanced glycation end products and toll-like receptors [47]. The elevation in fecal calprotectin implies neutrophil migration into the intestinal mucosa as a result of intestinal inflammation [48]. Similarly, the treatment of rats suffering from colitis with *Lactobacillus delbruekii* and *Lactobacillus fermentum* reduced the levels of fecal calprotectin compared with the colitic non-treated group [41]. Remarkably, our study revealed that the augmented role of MSPNPs in the reduction of FC levels was more prominent than that of free MSPs after the induction of colitis.

The expression levels of TLRs were upregulated as a consequence of colitis development in DSS-induced mice [49]. Similarly, the expressions of TLR2 and TLR9 were significantly upregulated in DSS-induced colitis. In contrast, MSPNP administration contributed to a significant reduction in TLR2 and TLR9, which is in accordance with the low levels of FC. These results demonstrate that MSPNPs exerted a more potent anti-inflammatory impact than free MSPs in experimentally induced colitic rats.

Pro-inflammatory cytokines are critical biomarkers which are released in the intestinal mucosa upon the hyper-activation of immune cells after the induction of IBD. It was confirmed that a selective blockade of these inflammatory mediators decreased neutrophil/macrophage migration, improving colitis progression [50]. Tumor necrosis factor alpha (TNF-α) and interleukin 6 (IL-6) play crucial roles in the pathophysiology of IBD [51,52]. They modify the mucosal immune system, change epithelial integrity and orchestrate neutrophil and macrophage infiltration and activation, which end colonic injury [52]. The pro-inflammatory cytokine levels implicated in this study were elevated in the serum of the colitic group, which is in harmony with previous research [22]. Additionally, the upregulated levels of pro-inflammatory cytokines such as *IL-18*, *IL1-B*, *IL-6* and *TNF-α* in colitic rats were downregulated in the MSPNP-treated group, which is suggestive of their potential role in treating IBD by blocking factors that injure the intestines and by augmenting factors that heal them. Correspondingly, beneficial *Lactobacillus* lowered the production of pro-inflammatory cytokines such as TNF-α and IL-6 [53,54]. Likewise, Niu et al. [55] showed that the oral administration of the *L. plantarum* strain (CAU1055) significantly diminished the expression levels of *TNF-α and IL-6.* Additionally, many studies have shown that a variety of lactic acid bacterial strains such as *Bifidobacterium bifidum* [56] and *Lactobacillus Gasseri* [57] prevent certain diseases associated with the gastro-intestinal tract. Moreover, *Lactobacillus acidophilus*, *Bifidobacterium longum* and *Ligilactobacillus salivarius* have been established to exert anti-inflammatory properties in many in vitro and in vivo studies by augmenting the level of anti-inflammatory cytokines while plummeting the production of inflammatory cytokines, including *TNF-α*, *IL-6* and *IL-1β* [58,59,60]. Similarly, a mixture of probiotics comprising *Lactobacillus acidophilus*, *Bifidobacterium* and *Enterococcus* reduced the colonic levels of TNFα when compared with the colitic non-treated group [61]. The observed downstream of inflammatory effectors was considered as a benefit of MSPNPs in the management of colitis and can be ascribed to their metabolic-factor release [62]. Additionally, the enhanced impact of MSPNPs as compared with free MSPs on the attenuation of induced colitis can be ascribed to their incorporation in nanoform, which plays a an crucial role in their survival and stability, thus boosting their mechanistic action in the GIT [63].

Probiotics and commensal bacteria were displayed to boost intestinal barrier integrity in a mouse model of colitis [64]. Cytokine release as a consequence of IBD progression is among the main causes that results in the downregulation of tight-junction-related genes [65,66]. Our findings show that rats that suffered from experimentally induced colitis exhibited a marked down-regulation of genes related to the integrity of tight junctions. Of note, the group orally administered with MSPNPs significantly ameliorated the expressions of these tight-junction-related markers, which indicates their protective role in colonic tissue. In accordance, the oral administration of *Bifidobacterium bifidum* enhanced intestinal epithelial tight junction gene expression [67]. Additionally, probiotics including *Bifidobacterium*, *Enterococcus*, *Lactobacillus* and *Lactococcus lactis* were shown to stimulate intestinal epithelial barrier integrity through the modulation of tight-junction-related gene expression [68,69]. The treatment of animal models of colitis with probiotic bacteria confers protecting effects against TJ barrier damage induced by numerous factors, such as pro-inflammatory cytokines, pathogen infection and oxidative stress [70]. Additionally, compared with the colitic group, the expressions of tight-junction-related genes including *JAM*, occludin and claudin were significantly reduced in the group treated with a mixture of probiotic bacteria including *Bifidobacterium*, *Lactobacillus acidophilus* and *Enterococcus* [61]. MSPNPs were shown to enhance intestinal integrity in DSS-induced colitis by augmenting the gene expressions of ocludin, JAM and ZO-1, unlike in the colitic non-treated group, which is in agreement with Mennigen et al. [71].

NLR family member NLRP3 is a critical regulator of intestinal homeostasis, as its activation could play a protecting role in sustaining the balance of gut immunity; alternatively, if uncontrolled, its over-activation might result in the damage of tissues [72]. Additionally, NLRP3 inflammasome has been investigated relatively to having a role in the pathogenesis of ulcerative colitis [73], as it is responsible for activating the expression of caspase-1, producing IL-1β and IL-18 and starting the inflammatory process [74]. Although the activation of NLRP3 inflammasome is largely beneficial to the host defense during infections and metabolic processes, the over-production of IL-1β and IL-18 results in sterile inflammation, which can increase the risk of developing metabolic and autoinflammatory diseases among patients. Moreover, IL-1β is an important pro-inflammatory cytokine, and its production is also controlled by caspase-1 [75]. Additionally, caspase-1 is accountable for the processing and secretion of IL-18 and is required for pyroptosis, which is a form of cell death detectable during necrosis (inflammation and cytokine release) [76]. The activation of inflammasomes can trigger caspase-1 and further production of IL-1β [77]. Recently, probiotics were indicated to play an effective role in controlling the activity and expression of inflammasome, as they are concerned in the caspase-1-dependent processing of both IL-1β and an associated pro-inflammatory cytokine, IL-18 [78]. Herein, our data showed that NLRP3 inflammasome was upregulated in the group that suffered from DSS-induced colitis, which is consistent with Liu et al.’s findings [79]. In contrast, NLRP3 inflammasome activation was downstreamed more prominently by the oral administration of MSPNPs, which is consistent with the downregulation of caspase-1, IL-1β and IL-18, in agreement with Yin et al. [80]. Teixeira et al. [81] stated that lactobacilli can reduce caspase-1 expression and maturation. Similarly, microbe-derived antioxidants from *Bacillus subtilis* and *Lactobacillus* downregulated the gene expressions of *IL-1β*, *IL-18* and *NLRP3* in mice [82]. The effective therapeutic effects of MSPNPs in colitic rats may be ascribed to their nanodelivery system that boosts their viability and resistance under simulated gastrointestinal conditions, thus augmenting their anti-inflammatory properties in rats model of IBD [83].

Previous trials have described that changes in the gut microbiota can play a critical role in the progress of DSS-induced colitis [84,85]. From this viewpoint, novel insights for treating IBD aiming at modifying gut microbiota have gained much attention [32]. *Bifidobacterium* displayed its impacts by sustaining the intestinal barrier that protects colonic tissues against ulcerative factors and inflammation [86]. *Lactobacillus* has a significant role in attenuating IBD. Moreover, the negative association between *Lactobacillus* counts and active IBD patients suggested their intrinsic pharmabiotic function in the attenuation of IBD by modifying the gut homeostasis of microbiota [87]. Herein, the oral administration of MSPNPs alleviated the microbial imbalance detected after DSS induction, whereby colon microbiota were directed toward beneficial ones, and the pathogenic population was reduced, unlike in the DSS-induced non-treated group. Additionally, the incorporation of multi-strain probiotics into nanoparticles evidently improved this function. In accordance with our findings, a decrease in *Bifidobacterium* was previously identified in ulcerative colitis patients [88]. In accordance with this, the beneficial effects on colitis mouse models were found following the oral administration of a mixture of probiotics comprising *Lactobacillus* and *Bifidobacterium* [34]. This function of MSPNPs may be attributed to their role in attenuating the oxidative damage that accompanies the induction of colitis, which is in agreement with [89]. *Firmicutes* have several cellulolytic organisms that are valuable for cellulose degradation [90]. Additionally, *bacteroides* are capable of decomposing organic matter and enhancing the innate immune response [91]. Furthermore, the *Bacteroidetes*-to-*Firmicutes* ratio is an index to assess the gut microbial composition [92]. After the induction of colitis, the group that received MSPNPs had a prominent shift to an increased *Firmicutes*-to-*Bacteroidetes* ratio that was responsible for attenuating inflammatory bowel disease development [1,93]. The augmented role of MSPNPs in modifying gut microbial communities resulted from their effective protection and long-lasting survivability in the gastrointestinal tract, which is in agreement with [94].

After IBD induction, the colon histological examination was in harmony with the above results, as the colon epithelial architecture exhibited complete destruction with extensive infiltration of inflammatory cells and damage of colon crypts. These histological alterations were in accordance with previous findings reported for DSS-induced colitic models of IBD [6,95]. Moreover, the infiltration of leukocytes into the lamina propria and submucosa with prominent ulceration signify an acute activation of the local immune responses after the induction of IBD [37,96]. In contrast, the groups treated with free MSPs and MSPNPs displayed a significantly lower damage of the histoarchitecture of the colon. Moreover, the recovery and restoration of colonic tissues were more prominent in the group orally administered with MSPNPs than with free MSPs. *Lactobacillus* spp. reduced leaky gut and inflammation, which could clarify the slightly enhanced function of free-Lactobacillus treatment [97]. Similarly, rats treated with *Bacillus amyloliquefaciens* loaded with nanoparticles showed less sever histopathological changes in the colon [28].

## 5. Conclusions

This study shows that MSPNPs were more effective in ameliorating DSS-induced colitis in an experimental model than free MSPs. The favorable actions of MSPNPs could be related to their incorporation in the nanodelivery system, which protects them and promotes targeted delivery. In addition, the role of MSPNPs in modulating the inflammatory mediators and NLRP3 inflammasome and caspase-1 gene expressions, together with upregulating tight-junction-related genes, was more prominent than that of the free-MSP group. Finally, the current study provides unique prospective insights that augment the potential role of newly formulated probiotic-strain-loaded nanoparticles in attenuating DSS-induced colitis, an experimental model of IBD.

## Figures and Tables

**Table 1:** Assessment by disease activity index (DAI).

	Weight Loss (%)	Stool Consistency	Gross Bleeding
Scores	0 = None	0 = Normal	0 = Negative
	1 = 0.1–5%		
	2 = 5–10%	2 = Loose stool	2 = Hemoccult
	3 = 10–20%		
	4 = >20%	4 = Diarrhea	4 = Gross bleeding

**Table 2:** Primer sequences utilized for the qRT-PCR analysis of targeted gene expression.

Target Gene	Primer Sequence (5′–3′)	Accession No./Reference
OCCLUDIN	F-CTGTCTATGCTCGTCATCG R-CATTCCCGATCTAATGACGC	NM-031329
*JAM*	F-GCTCAGCC ATACAGCAAATCC R-GGGAGTCGGGCAAT CATCAG	NM_017232
*MUC-2*	F-CAGAGTGCATCAGTGGCTGT R-CCCGTCGAAGGTGATGTAGT	XM_039101270.1
*MUC-5*	F-AACTCTGCCCACCACAAG R-TGCCATCTATCCAATCAGTCCAAT	XM_039101269.1
*ZO-1*	F-AGCGAAGCCACCTGAAGATA R-GATGGCCAGCAGGAATATGT	NM-001106266
*CLDN-2*	F-TCTGGATGGAGTGTGCGAC R-AGTGGCAAGAGGCTGGGC	NM-001106846
*FABP-2*	TGACGATCACACAGGAAGGA CCAGAAATCTCTCGGACAGC	XM_032897378.1
*IL-1β*	F-TGACAGACCCCAAAAGATTAAGG R-CTCATCTGGACAGCCCAAGTC	NM_031512.2
*IL-6*	F-CCACCAGGAACGAAAGTCAAC R-TTGCGGAGAGAAACTTCATAGCT	NM_012589.2
*IL-18*	F- ATGGCTGCCATGTCAGAAGA R-TTGTTAAGCTTATAAATCATGCGGCCTCAGG	XM_039080945.1
*IL-10*	F-GCCCAGAAATCAAGGAGCATT R-CAGCTGTATCCAGAGGGTCTTCA	L02926.1
*TNFα*	F-CAGCCGATTTGCCATTTCA R-AGGGCTCTTGATGGCAGAGA	L19123.1
*TLR2*	F-CGCTTCCTGAACTTGTCC R-GGTTGTCACCTGCTTCCA	XM_008761102.3
*TLR9*	F-CCGAAGACCTAGCCAACCT R-TGATCACAGCGACGGCAATT	XM_032911318.1
*Caspase-1*	F-GTGTTGCAGATAATGAGGGC R-AAGGTCCTGAGGGCAAAGAG	NM_012762.3
*NLRP3 inflammasome*	F-CCAGGGCTCTGTTCATTG R-CCTTGGCTTTCACTTCG	XM_039085397.1
β-actin	F-CGCAGTTGGTTGGAGCAAA R-ACAATCAAAGTCCTCAGCCACAT	V01217.1
*GAPDH*	F-TGCTGGTGCTGAGTATGTCG-3′ R-TTGAGAGCAATGCCAGCC-3′	NM_017008
*Bacteroides* spp.	F-GAG AGG AAG GTC CCC CAC R-CGC TAC TTG GCT GGT TCA G	Layton, McKay, Williams, Garrett, Gentry and Sayler [22]
*Lactobacillus*	F-AGCAGTAGGGAATCTTCCA R-CACCGCTACACATGGAG	[23]
*Firmicutes* spp.	F-GGAGYATGTGGTTTAATTCGAAGCA R-AGCTGACGACAACCATGCAC	Guo, Xia, Tang, Zhou, Zhao and Wang [24]
*Enterobacteriaceae*	F-CATTGACGTTACCCGCAGAAGAAGC R-CTCTACGAGACTCAAGCTTGC	Bartosch, Fite, Macfarlane and McMurdo [25]
*Bifidobacterium* spp.	F-GCG TCC GCT GTG GGC R-CTT CTC CGG CATGGT GTTG	Requena, Burton, Matsuki, Munro, Simon, Tanaka, Watanabe and Tannock [26]
*Bacillus* spp.	F-GCA ACG AGC GCA ACC CTT GA R-TCA TCC CCA CCT TCC GGT	Zhang, Chen, Yu, He, Yu, Mao, Wang, Luo, Huang and Cheng [27]

Junction adhesion molecule (*JAM*), mucin-2 (*MUC-2*), mucin-5 (*MUC-5*), zonula occludens (ZO-1), claudins (*CLDN-1*), fatty acid binding protein (FABP-2), Interleukin (*IL*)-*1β, IL-6, IL-18, IL-10*, tumor necrosis factor α (*TNFα*), toll-like receptors (*TLR2* and *TLR9*), pyrin domain-containing protein 3 (NLRP3) inflammasome.

**Table 3:** Cell survivability of free and nanoparticle-double-coated multi-strain probiotics (log CFU/mL) after treatment in activated gastric juice followed by activated intestinal juice with bile salt.

	No. of Survival Cells (CFU/mL)/min			
	30	60	90	120
Free *Lactobacillus acidophilus*	9.2 ± 0.21 × 10^9 a^	8.8 ± 0.21 × 10^9 ab^	8.3 ± 0.15 × 10^8 b^	6.1 ± 0.22 × 10^5 c^
*Lactobacillus acidophilus* double-coated with nanoparticles	9.8 ± 0.20 × 10^9 a^	9.1 ± 0.11 × 10^9 b^	8.4 ± 0.17 × 10^8 c^	5.8 ± 0.20 × 10^7 d^
Free *Bifidobacterium bifidum*	8.3 ± 0.10 × 10^8 a^	8.1 ± 0.25 × 10^7 b^	6.9 ± 0.3 × 10^6 c^	5.1 ± 0.21 × 10^6 d^
*Bifidobacterium bifidum* double-coated with nanoparticles	8.9 ± 0.17 × 10^9 a^	8.1 ± 0.13 × 10^9 ab^	8.9 ± 0.19 × 10^8 b^	7.5 ± 0.29 × 10^7 c^
Free *Bacillus amyloliquefaciens*	7.5 ± 0.22 × 10^9 a^	7.1 ± 0.3 × 10^8 b^	6.7 ± 0.24 × 10^8 bc^	6.1 ± 0.11 × 10^7 c^
*Bacillus amyloliquefaciens* double-coated with nanoparticles	8.6 ± 0.22 × 10^9 a^	7.9 ± 0.24 × 10^9 a^	7.1 ± 0.19 × 10^8 ab^	6.8 ± 0.22 × 10^8 b^

^a,b,c,d^ Means of the same rows with different letters were significantly different among groups (*p* < 0.05).

**Table 4:** Liver and kidney function tests and hematological indices of colitic rats orally administrated free or multi-strain-probiotic-loaded nanoparticles (MSPNPs).

Parameter	Control	DSS	DSS + MSPs	DSS + MSPNPs	*p*-Value	SEM
ALT (U/L)	46.23 ^c^	90.14 ^a^	50.33 ^b^	48.22 ^c^	0.02	0.23
AST(U/L)	23.29 ^d^	49.44 ^a^	29.86 ^b^	24.22 ^cd^	0.03	0.28
Urea	32.69 ^d^	50.25 ^a^	44.47 ^b^	35.51 ^c^	0.04	0.34
Creatinine	1.05 ^d^	3.3 ^a^	1.90 ^b^	1.63 ^c^	0.02	0.08
RBCs (×10^6^/μL)	11.33 ^a^	7.23 ^c^	9.25 ^b^	10.49 ^ab^	<0.001	0.13
Hb (g/dL)	12.93 ^a^	8.20 ^c^	8.75 ^c^	11.53 ^b^	<0.001	0.17
MPO (u/g tissue)	2.10 ^d^	14.36 ^a^	7.63 ^b^	4.27 ^c^	<0.001	0.29
CRP (mg/L)	0.85 ^d^	18.39 ^a^	10.36 ^b^	4.33 ^c^	<0.001	0.30
NO (nmol/g tissue)	178.36 ^c^	698.25 ^a^	386.32 ^b^	365.14 ^b^	0.03	0.39
TNF-α	25.36 ^d^	77.39 ^a^	32.36 ^b^	29.36 ^cd^	0.02	0.24
IFNγ	40.23 ^d^	83.39 ^a^	69.32 ^b^	46.31 ^c^	<0.001	0.34
IL-6	12.37 ^d^	36.98 ^a^	25.47 ^b^	20.36 ^c^	<0.01	0.09
IL-10	280.36 ^c^	789.36 ^a^	546.32 ^b^	569.36 ^b^	<0.01	0.43

ALT, alanine transaminase; AST, aspartate transaminase; RBCs, red blood cells; Ht, hematocrit; Hb, hemoglobin; MPO, myeloperoxidase; CRP, C-reactive protein; NO, nitrous oxide; TNF-α, tumor necrosis factor alpha; IFNγ, Interferon gamma; IL, interleukin. Mean values with different letters in the same row differed significantly at *p* < 0.05. SE, standard error. ^a,b,c,d^ Means of the rows with different letters were significantly different among groups (*p* < 0.05). Non-colitic group: Control (rats were orally gavaged with PBS). Colitic groups including: DSS (rats were orally gavaged with dextrane sodium sulphate), DSS + MSPs (rats were orally gavaged with DSS and multi-strain-probiotics (MSPs) at the level of 1.0 × 10^10^ CFU/kg in 1 mL of PBS/rat/day for 14 days), DSS + MSPNPs (rats were orally gavaged with DSS and multi-strain-probiotic-loaded nanoparticles (MSPNPs) at the level of 1.0 × 10^10^ CFU/kg in 1 mL of PBS/rat/day for 14 days); all groups were orally gavaged with 5% DSS.

## Data Availability

The data presented in this study are available upon request from the corresponding author.
